# Negative Body Image Associated with Changes in the Visual Body Appearance Increases Pain Perception

**DOI:** 10.1371/journal.pone.0107376

**Published:** 2014-09-11

**Authors:** Michihiro Osumi, Ryota Imai, Kozo Ueta, Satoshi Nobusako, Shu Morioka

**Affiliations:** 1 Department of Neurorehabilitation, Graduate School of Health Science, Kio University, Nara, Japan; 2 Neurocognitive Rehabilitation Center, Setsunan General Hospital, Osaka, Japan; Birkbeck, University of London, United Kingdom

## Abstract

Changing the visual body appearance by use of as virtual reality system, funny mirror, or binocular glasses has been reported to be helpful in rehabilitation of pain. However, there are interindividual differences in the analgesic effect of changing the visual body image. We hypothesized that a negative body image associated with changing the visual body appearance causes interindividual differences in the analgesic effect although the relationship between the visual body appearance and analgesic effect has not been clarified. We investigated whether a negative body image associated with changes in the visual body appearance increased pain. Twenty-five healthy individuals participated in this study. To evoke a negative body image, we applied the method of rubber hand illusion. We created an “injured rubber hand” to evoke unpleasantness associated with pain, a “hairy rubber hand” to evoke unpleasantness associated with embarrassment, and a “twisted rubber hand” to evoke unpleasantness associated with deviation from the concept of normality. We also created a “normal rubber hand” as a control. The pain threshold was measured while the participant observed the rubber hand using a device that measured pain caused by thermal stimuli. Body ownership experiences were elicited by observation of the injured rubber hand and hairy rubber hand as well as the normal rubber hand. Participants felt more unpleasantness by observing the injured rubber hand and hairy rubber hand than the normal rubber hand and twisted rubber hand (p<0.001). The pain threshold was lower under the injured rubber hand condition than with the other conditions (p<0.001). We conclude that a negative body appearance associated with pain can increase pain sensitivity.

## Introduction

“Pain” is a subjective experience. It not only occurs when a peripheral organ is damaged but also is easily influenced by the central nervous system [1]. Therefore, pain is modulated by attention [2], expectation [3], emotion [4], social factors [5], and emotional facial expressions [6], Recently, the perceived pain level was modulated by introducing a pain stimulus and showing visual information, such as various facial expressions or pictures of a romantic partner at the same time [7]. Also, according to a report by Longo et al. [8], individuals felt less pain when they looked at their own bodies than when they looked at an object. This analgesic effect had been identified by nociceptive laser-evoked potentials recorded by an electroencephalogram [8]. Also, it was reported that brain activity-related pain was lower when looking at a body than an object [9]. More recently, it has been said that body ownership can increase the analgesic effect of looking at the body. For example, Romano et al. reported that during states of illusory self-identification with the avatar, looking at the body was effective in modulating physiological responses to painful stimuli [10]. Also, Martini et al. reported that the pain threshold was increased when looking at a virtual body, which participants felt was their own body, than looking at an oblique cylinder [11]. Similarly, there have been reports of the modulation of pain perception by looking at a rubber hand that was felt to be one's own hand. Mohan et al. reported that the same activity did not induce analgesia [12]. However, Hegedus et al. found that looking at a rubber hand that was felt to be one's own hand induced analgesia, which identified and remedied the problem of how to give pain stimulation experienced in Mohan's experiment [13]. In this way, it was clarified that body ownership can increase the analgesic effect of looking at the body. On the other hand, some studies reported that looking at a distorted body image (in size and color) had an even greater pain modulating effect than a regular body image [14], [15]. Mancini et al. reported that individuals felt less pain when their own bodies appeared large in a concave mirror, but felt increased pain when their bodies appeared shrunken in a convex mirror [14]. Romano et al. reported that looking at an enlarged version of their own body decreased physiological responses to painful stimuli [16]. Martini et al. used a virtual reality system that colored the visual body appearance red or blue. The participants felt stronger pain when their body appeared red while they felt less pain when the body appeared blue [15]. Such visual body image-based interventions have been applied to patients with chronic pain who have complex regional pain syndrome, phantom limb pain, chronic back pain, and osteoarthritis [17]–[20]. For example, phantom limb pain was decreased by individuals observing their shrunken body in a funny mirror. Of note, however, is that manipulation of visual body images has not produced reliable intervention effects. For example, Preston et al. reported that the appearance of extended fingers had an analgesic effect in some osteoarthritis patients while the appearance of shortened fingers had the same effect for others [20]. Although the pain and edema in complex regional pain syndrome were increased by looking at one's own body magnified [17], in other cases the same visual distortion led to pain reduction [14]. Results varied when summarizing reports of the analgesic effect of looking at a distorted body appearance. However, the reasons have not been shown. Mirror visual feedback by looking at one's own body is one of the rehabilitation tools. Although mirror therapy is effective for pain [21]–[23], there have been reports of adverse effects, for example, increased pain [24], [25], Hagenberg et al. reported that “emotional reaction” is the most common adverse effect of mirror visual feedback [26]. Osumi et al. also reported that individuals who had a negative emotion toward to a distorted body appearance felt increased pain while looking at a distorted body appearance [27]. In this way, negative emotion toward one's body image (negative body image) is one factor in preventing an analgesic effect by looking at a distorted body appearance. However, there are different forms of a negative body image. A negative body image is now believed to include anxiety arising from looking at one's own body, embarrassment from being seen by others, and recognition of one's own body not conforming to an established idea of a desirable appearance [28]. Regarding anxiety arising from looking at one's own body, looking at one's injured body increased brain activity-related pain [29], [30]. As to embarrassment from being seen by others, in experiments involving individuals with bulimia nervosa there was increased brain activity-related pain, for example, that of the anterior cingulate cortex or medial prefrontal cortex when they viewed their own fat bodies [31]. Concerning one's body not conforming to the established idea of what a body should look like, discomfort with simulating sensorimotor incongruence evoked brain activity-related pain [32], [33]. Based on these studies, we hypothesized that the three species of negative body image would increase physical pain because they could increase brain activity-related pain. But it is considered that those who have a negative body image are sensitive to pain. The objective of this study therefore was to identify specific types of negative body image that would worsen a perceived pain level.

## Materials and Methods

### Ethics Statement

The experimental protocol was approved by the Kio University Ethics Committee (approval number: H24-19) and the study protocol conformed to the Declaration of Helsinki. Participants gave written consent to participate after receiving an explanation of the procedures involved.

### Participants

A total of 25 healthy right-handed students (9 males, 16 females; mean age, 21.61 years; SD, 0.56) participated in this study. Prior to the experiment, the experimental procedure was explained to them. The purpose of the experiment, however, was not explained to prevent the participants from having any bias.

### Rubber Hand Illusion

**Figure 1:**
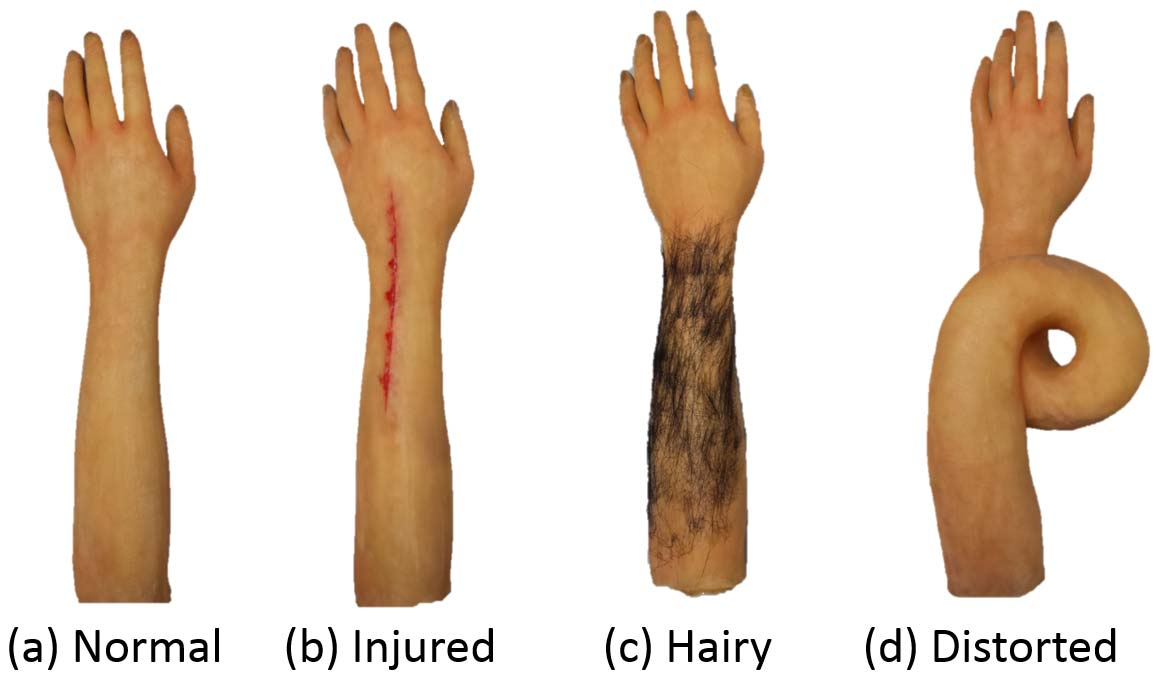
Rubber hands used in the experiments. We used 4 rubber hands that represented the following conditions: (a) did not evoke a negative body image, (b) evoked a pain-related negative body image, (c) evoked a negative body image due to non-conformance of a socially accepted appearance, and (d) evoked a negative body image due to non-conformance of the concept of a normal body.

A rubber hand illusion was used to evoke a negative body image associated with one's own body. The rubber hand illusion is a body ownership illusion in which individuals start to feel that the fake hand is their own hand when a hidden real hand and a rubber hand placed in front of them are touched simultaneously [34]. In recent years, it has been used to let individuals experience a wide variety of perceptions. For example, a rubber hand with no fourth finger allows individuals to experience a phantom fourth finger [35]. Also, a black rubber hand allows non-black individuals to experience what it is like to have dark skin [36]. For this study, special rubber hands designed to evoke a negative body image were created. Study participants were then led to develop a sense of ownership over these hands so that they would experience a negative body image about their own bodies. In this study, “rubber hand” refers to both the forearm and hand. Examples of a negative body image include anxiety that arises when individuals see what has happened to their bodies; inferiority that arises when they compare themselves with others; embarrassment that arises when they are seen by others; and an emotion that arises when their appearance does not conform to an established idea of a desirable physical appearance [26]. For the purpose of this study, the following types of rubber hands were created (Fig. 1): (a) a normal rubber hand that would not make the participants feel uncomfortable (normal rubber hand: Normal); (b) an injured rubber hand to make the participants feel uncomfortable by the sight of an injury (injured rubber hand: Injured); (c) a hairy rubber hand to make the participants feel uncomfortable about being seen by others (hairy rubber hand: Hairy); and (d) a distorted rubber hand to make the participants feel uncomfortable because their body does not conform to the established idea of what a body should look like (distorted rubber hand: Distorted). In addition, for each type of rubber hand, we provided both illusion and no illusion conditions.

**Figure 2:**
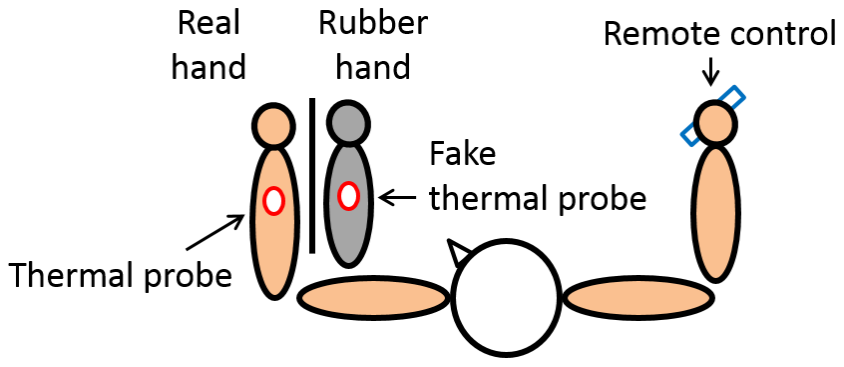
Experimental setting for pain threshold measurement. A thermal probe was placed on the participant's forearm and a fake thermal probe was placed on the forearm of the rubber hand. The participant was instructed to stare at the rubber hand during the pain threshold measurement. The experimenter increased the temperature of the stimulus by 1°C per second. The participant used a remote control in his/her right hand to stop the temperature from increasing any further the moment pain was felt. The temperature was recorded as the pain threshold.

Participants sat on a chair and placed the left index finger on a spot marked on the table. On the table, a board was installed parallel to the sagittal plane to keep participants from seeing their own hand. In the illusion condition, a rubber hand was then placed on the table with its left index finger 15 cm away to the right of the real left index finger and also vertically aligned with the left armpit when seen from the front (Fig. 2). In the no illusion condition, the posture of the rubber hand was slightly incongruent with the real hand, though anatomically possible (rotated by 20–30°) in order to maximize the reduction of the illusion. The method of Hegedüs et al. was used as a reference to setting this no illusion condition [24]. In both the illusion and no illusion conditions, a bath towel was put over the proximal end of the rubber hand as well as the left arm and shoulder of participants so they could not see how the real and fake hands were arranged in this area. Participants were instructed not to move the left hand and to keep looking at the rubber hand during the experiment.

### Pain Stimulation Device and Pain Threshold Measurement

Pain thresholds were measured by applying stimuli to the back of the left forearm (10 cm from the left wrist) by a thermal stimulator (UDH-105, UNIQUE MEDICAL, Tokyo, Japan). The thermal probe was 20 mm in size and was directly placed on the measurement point. Measurement was conducted in accordance with the study by Yarnitsky et al. [37]. The thermal stimulus started at 32°C with a 1°C increment per second. The temperature at which the participant felt the stimulus as painful was recorded as the pain threshold. Participants were instructed to press the switch on the remote control in the right hand the moment they felt pain so that the temperature would not be increased further (Fig. 2). Note that thermal stimuli were introduced a few times to an area (center of the back of a hand) not subject to the threshold measurement so that participants could become sufficiently accustomed to the pain caused by thermal stimuli prior to the experiment.

### Procedure

**Figure 3:**
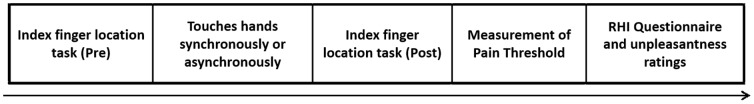
Experimental procedure. The experiment consisted of 4 sequences of experimental steps for each of the 8 conditions presented by the rubber hands. Each sequence was designed to include an index finger location task, pain threshold measurement, a rubber hand illusion questionnaire, and unpleasantness rating.

This procedure involved 8 different conditions: Normal rubber hand (illusion/no illusion), Injured rubber hand (illusion/no illusion), Hairy rubber hand (illusion/no illusion), and Distorted rubber hand (illusion/no illusion). These conditions were randomized across subjects. The experiment consisted of 4 sequences of experimental steps for the 8 conditions presented by the rubber hands. Each sequence was designed to include an index finger location task, pain threshold measurement, and a rubber hand illusion questionnaire. Fig. 3 shows a diagram of the sequence of the experiments.

All sequences were performed identically and included the index finger location task to examine the effectiveness of the illusion of body ownership over the rubber hand and the rubber hand illusion questionnaire (RHI Questionnaire).

The first step of a sequence was the index finger location task. An acrylic board and cloth were used to prevent participants from seeing the real hand and rubber hand. Then, the experimenter who was seated in front of the participant slowly moved experimenter's index finger from the left side of the participant (20 cm to the left of the real index finger) toward the midline. Participants were instructed to verbally inform the experimenter when they thought that the experimenter's index finger was above their left index finger. The experimenter recorded the reported position.

The second step was a rubber hand illusion. In the illusion condition, the illusion was created using 2 paintbrushes, stimulating both the rubber hand and the real hand for 5 minutes on the same locations and at the same time. In the no illusion condition, both the rubber hand and the real hand were stimulated for 5 minutes on different locations and at different times. The participant was instructed not to move his/her left hand and to keep looking at the rubber hand.

After the rubber hand illusion or no illusion task, the third step was another index finger location task, which was performed in the same way as the first index finger location task. The distance between the participant's reported location of the index finger before and after the rubber hand illusion was recorded as the proprioceptive drift and used in the analysis as the perceived effectiveness of the body ownership illusion. A larger value meant that the location of the real index finger reported by the participant was closer to that of the rubber hand. In other words, a larger value meant that the rubber hand illusion was more effective [38].

The fourth step was the pain threshold measurement on the back of the participant's hidden left forearm. A mock probe was placed on the rubber hand in order to make the participant feel that the pain came from the rubber hand (Fig. 2). In the experiment, the pain threshold was measured 4 times with a 1-minute interval between measurements. The number of measurements of pain threshold and the time of the interval were determined by reference to an experiment devised by Mancini [14] who used a pain stimulation device similar to our device. The average of the 4 measurements was used as the pain threshold value in the analysis.

After the pain threshold measurement, the final step was administration of the RHI Questionnaire and ratings of unpleasantness. The RHI questionnaire items were created based on the questionnaire developed by Longo et al. [39]. The participant used a 7-point Likert scale from −3 (strongly disagree) to +3 (strongly agree) to rate the following 4 statements: “When a rubber hand was stroked by a paintbrush, it felt as if the paintbrush stroked the same place on my hand,” “When a rubber hand was stroked by a paintbrush, it felt as if the paintbrush touched my hand,” “It felt as if the rubber hand was my own hand,” and “It felt like my left hand moved to the right (towards the rubber hand).” The RHI Questionnaire was administered under both the illusion and no illusion conditions. The ratings given to the 4 questions were averaged for each condition. The obtained values were then compared. Also, the participants rated 3 kinds of unpleasantness using a numerical rating scale from 0 (no unpleasantness) to 10 (worst possible unpleasantness). The numerical rating was done only for the illusion condition. The participants rated “unpleasantness associated with injury,” “unpleasantness associated with embarrassment” and “unpleasantness associated with nonconformance to the ideal body shape” for the 4 kinds of rubber hand.

## Statistical Analysis

Because the values for the perceived effectiveness of the illusion, proprioceptive drift, and pain threshold did not have normal distribution in Shapiro-Wilk tests, two-factorial ANOVA was not used. So, the perceived effectiveness of the illusion, proprioceptive drift, and pain threshold were analyzed using the Friedman test across rubber hand conditions under both illusion and no illusion conditions. In both of these cases, Wilcoxon signed-rank tests were used for post hoc analyses and the Bonferroni correction was used to adjust the p-values obtained in the post hoc analyses. In the present study, results using the illusion condition were compared among 4 rubber hand conditions; in addition, results from each rubber hand condition were compared between illusion and no illusion conditions. A total of 10 comparisons were made, and the significance level was set at P<0.005. Unpleasant feelings evoked by the illusion were compared across rubber hand conditions for each item separately using the Friedman test, with Wilcoxon signed-rank tests used for post hoc analyses. Bonferroni correction was used for the 4 items and the significance level was set at P<0.0083. SPSS ver. 17.0 (SPSS, Chicago, IL, USA) was used for statistical processing.

## Results

Table 1 shows the proprioceptive drift, perceived effectiveness of the illusion and pain threshold for each condition.

**Table 1:** Value of drift (SE), subjective degree of illusion (SE), and pain threshold (SE) under each condition.

	Normal		Injured		Hairy		Distorted	
	illusion	no illusion	illusion	no illusion	illusion	no illusion	Illusion	no illusion
Value of drift (cm)	2.25 (0.22)	0.19 (0.07)	2.58 (0.31)	0.10 (0.70)	2.41 (0.32)	0.14 (0.06)	1.16 (0.32)	0.19 (0.05)
Subjective degree of illusion	2.27 (0.13)	-2.54 (0.12)	2.02 (0.16)	-2.48 (0.16)	1.92 (0.16)	-2.64 (0.11)	0.8 (0.21)	-2.68 (0.11)
Pain threshold (°C)	43.11 (0.32)	43.37 (0.29)	42.01 (0.40)	43.43 (0.32)	43.08 (0.28)	43.35 (0.29)	42.92 (0.33)	43.41 (0.29)

### Proprioceptive Drift

**Figure 4:**
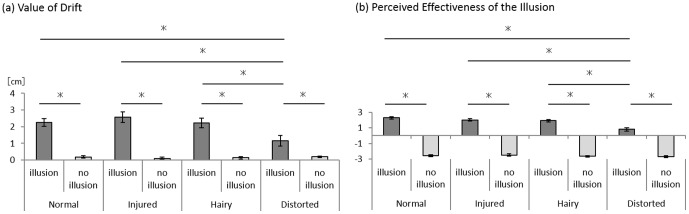
Value of drift (a) and subjective degree of illusion (b) under each condition. The post-hoc test showed that the value of drift and perceived effectiveness of the illusion under the distorted condition were significantly lower than under the normal, injured, and hairy conditions. Error bars indicate ± SE. * p<0.005.

In comparisons of proprioceptive drift for the 4 rubber hand conditions under illusion conditions, the Friedman test showed a significant main effect (χ^2^ = 15.22, P = 0.002). Under the no illusion condition, the Friedman test showed no significant main effect (χ^2^ = 2.08, P = 0.557). The post-hoc test showed that the proprioceptive drift for the distorted condition under the illusion condition was significantly lower than for normal, injured, and hairy conditions under the illusion condition (p<0.001) (Fig. 4a and Table 1). For all 4 rubber hand conditions, there were significant differences between the illusion and no illusion conditions (p<0.001) (Fig. 4a and Table 1). These results mean that under the distorted conditions the distance between the actual and perceived locations of the index finger during the rubber hand illusion was shorter than for the other conditions.

### Perceived Effectiveness of the Illusion

In the comparison of perceived effectiveness of the illusion for the 4 rubber hand conditions under illusion conditions, the Friedman test showed a significant main effect (χ^2^ = 28.76, P<0.001). In the comparison under no illusion conditions, the Friedman test showed no significant main effect (χ^2^ = 4.35, P = 0.226). The post-hoc test showed that the perceived effectiveness of the illusion was significantly lower for the distorted condition under the illusion condition than for the normal, injured, and hairy conditions (p<0.001) (Fig. 4b and Table 1). Under the all rubber hand conditions, there were significant differences between the illusion and the no illusion conditions (p<0.001) (Fig. 4b and Table 1). These results mean that compared to other conditions, the rubber hand illusion under the distorted condition was perceived to be less effective.

### Unpleasant Feeling Evoked by the Illusion

**Figure 5:**
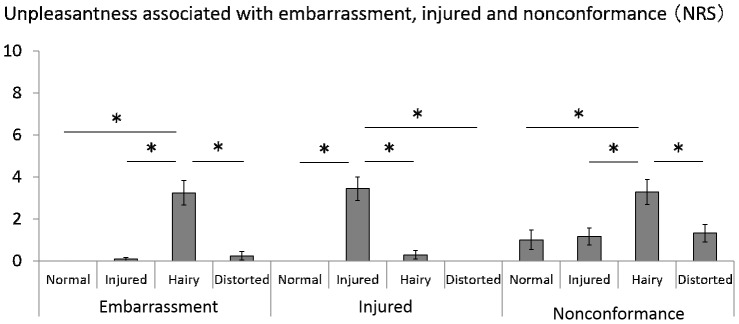
Unpleasantness associated with embarrassment, injury and nonconformance (NRS) under each condition. The post-hoc test showed that the hairy condition had a significantly greater impact than the normal, injured and distorted conditions for the item of unpleasantness associated with embarrassment. The post-hoc test showed that the injured condition had a significantly greater impact than normal, hairy and distorted conditions for the item of unpleasantness associated with injury while the hairy condition had a significantly greater impact than normal, injured and distorted conditions for unpleasantness associated with embarrassment. Error bars indicate ± SE. * p<0.0083.

In the item “unpleasantness associated with embarrassment”, the Friedman test and post-hoc test showed that the unpleasantness associated with embarrassment under the hairy condition was significantly stronger than for normal, injured and distorted conditions (X^2^ = 48.13, p<0.001; Hairy rubber hand: M = 3.24, SE = 0.59; Normal rubber hand: M = 0.00, SE = 0.00; Injured rubber hand: M = 0.08, SE = 0.08; Distorted rubber hand: M = 0.24, SE = 0.20) (Fig. 5). In the item of “unpleasantness associated with injury”, the Friedman test and the post-hoc test showed that unpleasantness associated with the injured condition was significantly stronger than for normal, hairy and distorted conditions (X^2^ = 49.29, p<0.001; Injured rubber hand: M = 3.44, SE = 0.56; Normal rubber hand: M = 0.00, SE = 0.00; Hairy rubber hand: M = 0.28, SE = 0.20; Distorted rubber hand: M = 0.00, SE = 0.00) (Fig.5). In the item “unpleasantness associated with nonconformance”, the Friedman test and post-hoc test showed that the unpleasantness associated nonconformance under the hairy condition was significantly stronger than for normal, injured and distorted conditions (X^2^ = 14.21, p<0.0083; Hairy rubber hand: M = 3.28, SE = 0.59; Normal rubber hand: M = 1.00, SE = 0.46; Injured rubber hand: M = 1.16, SE = 0.39; Distorted rubber hand: M = 1.32, SE = 0.41) (Fig. 5).

### Pain Threshold

**Figure 6:**
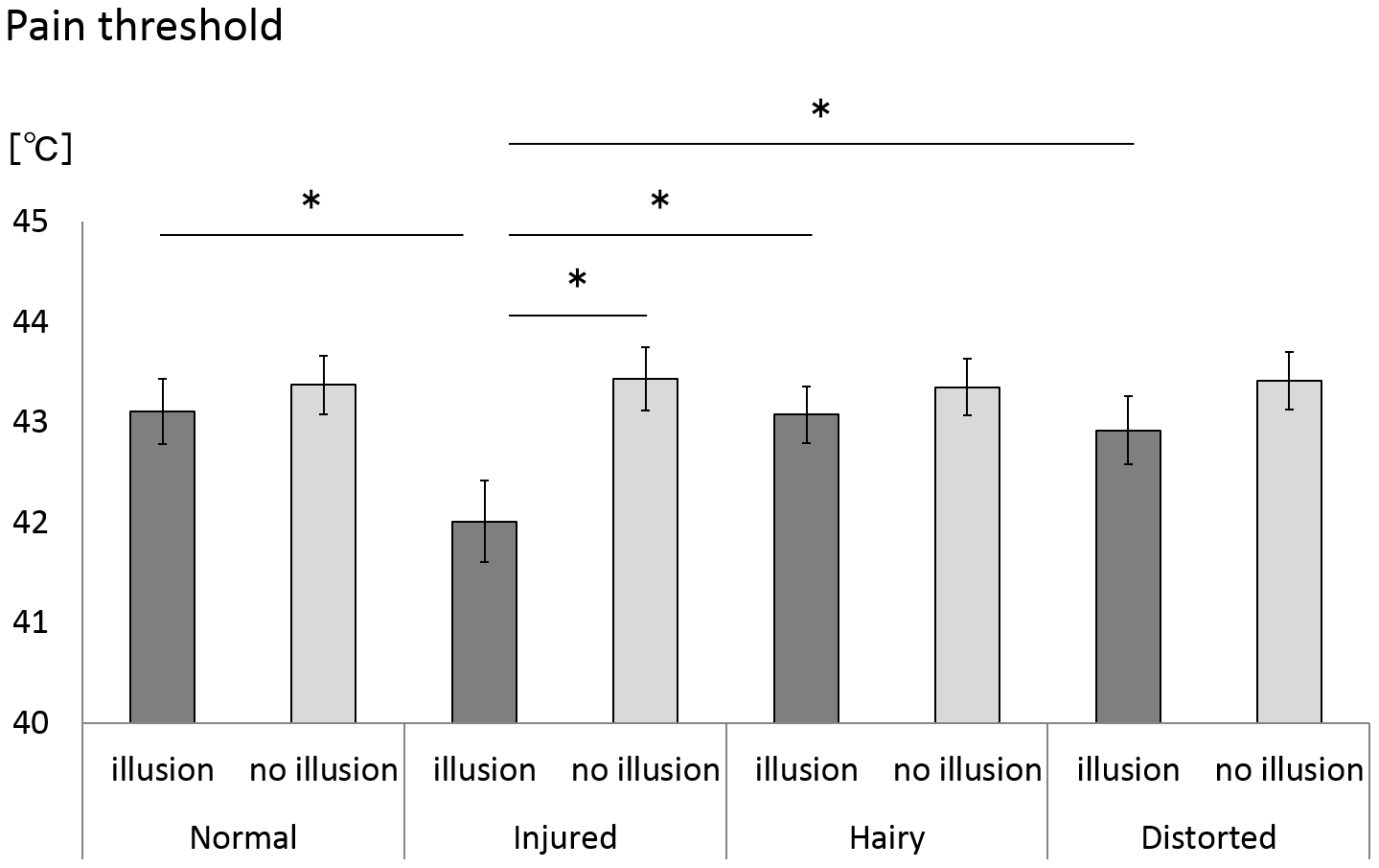
Pain threshold under each condition. The arm pain threshold was significantly lower under the injured condition than for the normal, hairy, and distorted conditions. Error bars indicate ± SE. * p<0.005.

In the comparison of the pain threshold for the 4 rubber hand conditions under illusion conditions, the Friedman test showed a significant main effect (X^2^ = 9.34, P = 0.025). Such a comparison under no illusion conditions showed that the Friedman test indicated no significant main effect (X^2^ = 1.72, P = 0.63) (Fig. 6 and Table 1). The post-hoc test showed that the pain threshold for the injured condition in the illusion condition was significantly lower than for normal, hairy and distorted conditions (p<0.005) (Fig. 6 and Table 1). Under the injured rubber hand condition, the Wilcoxon signed-rank test showed that the pain threshold with the illusion condition was lower than with the no illusion condition (P<0.005) (Fig. 6 and Table 1), but there were no significant differences under normal, hairy and distorted conditions (p>0.005).

## Discussion

In examining the influence of a negative body image on pain threshold through the rubber hand illusion, the injured and hairy rubber hands created the same level of body ownership illusion as the normal rubber hand. They also evoked a more negative body image than the normal rubber hand. As the result of unpleasant feelings evoked by the illusion (Fig. 5), participants felt specifically “unpleasantness associated with injury” in the injured rubber hand condition and participants felt specifically “unpleasantness associated with embarrassment” and “unpleasantness associated with nonconformance to the ideal body shape” with the hairy rubber hand condition. The experiment further showed that the pain threshold was lower when the participants were under the illusion of body ownership over the injured rubber hand compared to the illusion of body ownership over the other types of rubber hand.

### Effectiveness of the Illusion and Negative Body Image under Various Experimental Conditions

In the current experiments, the injured and hairy rubber hands created the same level of body ownership illusion as the normal rubber hand and also evoked a more negative body image than the normal rubber hand. The distorted rubber hand produced a less effective body ownership illusion than the other types of rubber hands and also failed to evoke any negative body image.

Many experiments have explored the necessary elements for creating a rubber hand illusion. According to these studies, a rubber hand must not only be stimulated at the same time and at the same location as the real hand but must also have corporeality [40], be anatomically identical to the real hand [41], and be arranged at the same angle as the real hand [42] to create an illusion. The distorted rubber hand was not anatomically identical to a real hand and also lacked corporeality, which would seem to have made it less likely for the distorted rubber hand to create an illusion. A recent study reported that a rubber hand could create a body ownership illusion without being influenced by its skin color as long as it was anatomically identical to a real hand [36]. The injured and hairy rubber hands had different skin appearances but were anatomically identical to a real hand. This seems to be the reason why these rubber hands created the same level of body ownership illusion as the normal rubber hand.

The illusion of body ownership over the injured and hairy rubber hands evoked a stronger unpleasant feeling than the normal rubber hand. A wide variety of psychological changes have been reported to occur during the process in which an illusion of body ownership over a rubber hand is created. For example, a 'bizarre and strange' feeling arose when an illusion of body ownership was created for a normal rubber hand [43]. Also, an illusion of body ownership over a rubber hand with a missing fourth finger evoked an unpleasant feeling such as pain [35]. Furthermore, there was a report of even a psychosocial change in which implicit racial biases held against people with dark skin turned into a positive feeling after individuals experienced an illusion of body ownership over a black rubber hand [44]. In the injured rubber hand condition, participants felt specifically “unpleasantness associated with injury”. With the hairy rubber hand condition, participants felt specifically “unpleasantness associated with embarrassment” and “unpleasantness associated with nonconformance to the ideal body shape”. The initial hypothesis for the study was that participants would feel only “unpleasantness associated with embarrassment” under the hairy rubber hand condition, but they also felt“unpleasantness associated with nonconformance to the ideal body shape”. We inferred that the reason for this result was that participants in this study were not actually hairy because participants included young adults and women. However, we could cause participants to feel“unpleasantness associated with the injury” and“unpleasantness associated with embarrassment” by feeling body ownership of the injured or hairy rubber hand.

The distorted rubber hand therefore failed to meet the prerequisite for being compared with other types of rubber hand because it was unlikely to create a body ownership illusion and did not evoke any unpleasant feeling. The injured and hairy rubber hands, on the other hand, were able to lead the participants to a psychological state of “negative body image about one's own body” by creating the same level of body ownership illusion as the normal rubber hand and also by evoking a stronger unpleasant feeling than the normal rubber hand.

### Pain Felt Under Each Experimental Condition

Under the normal rubber hand condition, there were no significant differences in pain threshold between the illusion and no illusion conditions. In a previous study, the effect of seeing a new dummy that embodied one's own body was unclear [11]–[13], [45]. In a previous study, it was unclear whether the effect of seeing embodied new dummy or virtual bodies [11]–[13], [45]. This difference in results is thought to be due to a difference in the experimental method. Among the various differences in experimental methods, the most important difference is whether there is“visual capture of pain”. When visual input predicts pain, there is anticipatory recruitment of the related brain region and enhanced pain perception [46]. Mohan et al. reported that the pain threshold did not change even with the rubber hand illusion [12]. They suggested that the heat probe on the rubber hand might have facilitated the “visual capture of pain”. This “visual capture of pain” evoked prediction of pain and enhanced sensitivity to pain [46]. In the present study, the dummy heat probe was placed on the rubber hand, and, as a result, seeing the dummy heat probe evoked prediction of pain. We thought that seeing the dummy heat probe was the reason the pain threshold did not differ significantly between illusion and no illusion conditions. However, it was very important that participants felt the illusion of body ownership and felt unpleasantness regarding a particular kind of rubber hand in order to achieve this present experimental objective. It was reported that synchronizing pain and visual stimuli evoked the rubber hand illusion as well as synchronizing tactile and visual stimuli [47]. From this study it can be considered that if there were no dummy heat probe on the rubber hand incongruity would occur between pain and visual stimuli and participants would not feel the illusion of body ownership of the rubber hand. Therefore, we used a dummy heat probe on the rubber hand when pain thresholds were measured.

The study found that the pain threshold was lower when the participant was under the illusion of body ownership over the injured rubber hand than when under the illusion of body ownership over other types of rubber hand. The initial hypothesis for the study was that the pain threshold would decrease when an unpleasant body image was evoked. What the study indicated, however, was that the pain threshold decreased only for the injured rubber hand even though both the hairy and injured rubber hand equally evoked an unpleasant feeling. Looking at one's own body is considered to produce an analgesic effect through the psychological effect of eliminating uncertainty about danger as well as any anxiety associated with it, thereby preventing pain sensitivity from becoming stronger [48]. Moseley et al., for example, instructed the participating patients with complex regional pain syndrome suffering from significant swelling to see their affected hands through binoculars and reported that the patients experienced increased pain under the illusion that the swelling had worsened [17]. Martini et al. used a virtual reality system to color the hands of the study participants red and instructed them to look at their colored hands. The participants felt that the pain worsened because that coloration reminded them of inflammation [15]. These examples suggest that the absence of negative perceptual experiences associated with pain is essential for the analgesic effect of looking at one's own body. In this study, both the injured and hairy rubber hands evoked an unpleasant feeling, but only the injured rubber hand resulted in worsening of pain. This result implies that, instead of a negative body image changing the pain threshold, pain worsens only when a pain-related negative body image is associated with a particular body part. In recent years, medical intervention for chronic pain patients through manipulation of a visual body appearance has been reported to be effective [17], [18], [20]. According to our experimental result, however, pain could worsen if a patient has pain-related unpleasant feelings toward the visual body image. Therefore, in clinical settings, it is desirable to provide a visual body appearance that reminds patients of reduced pain. For example, visual feedback that reminds a person of “cold” would be desirable for causalgia. Similarly, visual feedback that reminds a person of “stretching” would be desirable for pain accompanying stiffened limbs. Examination of these assumptions is our future clinical research theme.

This study has some limitations. First, emotional changes in the participants were measured only by a numerical rating scale, and it was not clear whether or not autonomic nervous responses actually occurred. It is necessary in the future to measure them, including skin conductance and heart rate. Second, only the sensory aspect of pain, or the pain threshold, was measured. Since pain also has cognitive and emotional aspects, the influence of pain on these aspects is a future research task. Third, the study did not clarify what kind of change in the central nervous system caused changes in pain. This is because brain activities were not measured by a brain function imaging device. This question should be clarified in the future through functional magnetic resonance imaging and/or electroencephalography. Lastly, all of the study participants were Japanese, so we did not consider the cultural background.

## Supporting Information

Table S1 **Outcomes of the study.**
(XLSX)
